# Peer evaluations of group work in different years of medical school and academic achievement: how are they related?

**DOI:** 10.1186/s12909-022-03165-5

**Published:** 2022-02-16

**Authors:** Zayar Linn, Yasura Tashiro, Kunimasa Morio, Hiroki Hori

**Affiliations:** 1 Department of Medical Education, Mie University Graduate School of Medicine, 2-174, Edobashi, Tsu city, Mie prefecture 514-8507 Japan; 2 Center for Medical and Nursing Education, Faculty of Medicine, Mie University, 2-174, Edobashi, Tsu city, Mie prefecture 514-8507 Japan; 3 College of Liberal Arts, Mie University, 2-174, Edobashi, Tsu city, Mie prefecture 514-8507 Japan

**Keywords:** Academic achievement, Book review sessions, Groupwork, Liberal arts education, Medical education, Peer evaluation, Problem-based learning, Teamwork

## Abstract

**Background:**

To develop the skills needed in health care teams, training communication and teamwork skills are important in medical education. Small group collaborative learning is one of the methods utilized in such trainings, and peer evaluation is suggested to be useful in reinforcing the effectiveness of group learning activities. In Mie University Faculty of Medicine, group work consisting of book review sessions of liberal arts education in the first grade and problem-based learning (PBL) sessions in preclinical years were conducted using the same peer evaluation system that included three domains: degree of prior learning, contribution to group discussion, and cooperative attitude. This study was conducted to determine the relationships among behaviors during group work and the academic achievement of medical students.

**Methods:**

With the data from a cohort of medical students in three consecutive academic years (*n* = 340), peer evaluation scores in groupworks of book review sessions, those in PBL sessions and paper test scores of preclinical years were analyzed. The correlations were analyzed with Spearman’s correlation coefficient, and the respective scores were compared by using the Wilcoxon signed-ranked test.

**Results:**

Significant correlations were observed among the evaluation scores of respective domains in group work and paper test scores. The degree of prior learning had the strongest relationship among the three domains (r_s_ = 0.355, *p* < 0.001 between book review sessions and PBL; r_s_ = 0.338, *p* < 0.001 between book review sessions and paper test score; r_s_ = 0.551, *p* < 0.001 between PBL and paper test score). Peer evaluation scores of respective domains were found to be significantly higher in PBL.

**Conclusion:**

Medical students maintained their groupwork behaviors to some extent from early school to preclinical years. Those behaviors were positively related to their academic achievement in the later years of the medical education curriculum. Our study highlighted the importance of the early introduction of group work. The results will be useful to motivate medical students to put more effort into group work.

**Supplementary Information:**

The online version contains supplementary material available at 10.1186/s12909-022-03165-5.

## Introduction

Current health care services are trying to provide comprehensive care, in which interdisciplinary teams play a major role. To provide efficient team members, teamwork skill trainings are inevitable in medical education. Communication and teamwork skills have become important learning goals in training medical professionals [1]. Moreover, the cooperative attitude of a student is essential for developing social skills and to be successful in college [2]. Small group learning is one of the methods utilized to help the development of teamwork, collaboration, and communication skills for practical situations [3].

In Japanese universities, liberal arts education programs have focused on small group learning [4]. The Japanese education system after World War II was inspired by that of the United States. However, the meaning of “liberal arts” in Japan is diverse and quite different from the original [5, 6]. In Japan, liberal arts education is also referred to as “general education” or “common education”. It is programmed mainly for first-grade undergraduate students before their professional education. In this study, “liberal arts education” is defined as a general education program scheduled in the first grade for all undergraduate students. The program mainly focuses on developing generic skills: basic skills on learning, reading, writing, communication, teamwork, presentation, language, information processing, and so on. To provide basic knowledge and skills required for medical professionals, liberal arts education is considered a helpful preparation for medical education [7, 8]. In Japan, some liberal arts education programs include problem-based learning (PBL) in small groups [9, 10]. Small group PBL is also widely implemented in many Japanese medical schools for developing teamwork and communication skills as well as critical and logical thinking [11]. The model core curriculum for medical education in Japan also highlights the development of communication skills for students [12]. For developing teamwork, collaboration and communication skills, small group learning plays a pivotal role in both liberal arts and medical education.

In the clinical environment, group work or teamwork skills, which reflect Miller’s pyramid level “does”, can be assessed by using tools for work-based performance assessment [13, 14]. However, for undergraduate medical students, especially in the programs for general education, assessment of group work behaviors needs to be well defined. Health professional trainings use various assessment methods to reinforce the effectiveness of group learning activities. For improving students’ communication and teamwork skills, peer evaluation or peer assessment is suggested to be useful if it is carefully designed to avoid social issues [15]. Peer evaluation can help medical students improve the quality of learning and develop reflective skills and concepts on performance standards [15–18]. Peer evaluation in a collaborative learning environment is suggested to be helpful for the development of professional behavior and for students’ engagement in learning [19, 20]. Early introduction of peer evaluation in medical education may encourage students to practice and accept critical evaluation [18]. The evaluations can be made more reliable by proper training for students and through teacher assistance [15, 21]. Implementing an appropriate assessment or an evaluation system to improve students’ learning behaviors requires much time and effort. Although the importance of peer evaluation is highlighted, there is limited information about the relationship of evaluations in different years of medical school, especially from general to professional education.

This study was conducted at Mie University, a typical Japanese national university with five faculties of social and natural sciences. All undergraduate students in this university learn liberal arts education together in their first grade. This education program of liberal arts is for developing students with autonomous active learning. It aims to train persons that can appropriately respond to globalization, various cultures, and different societies [22]. Book review sessions in liberal arts education are designed to foster students’ logical and critical thinking, communication, and problem-solving abilities. These sessions, which are conducted in small groups, aim to cultivate students’ attitudes and social ethics as members of a society [23]. A peer evaluation system, which contains three domains, is used to assess students’ groupwork behaviors in these sessions. The evaluation system was inspired from that of the PBL program of medical school. One of our previous studies found that students could reliably evaluate their peers in group book review sessions using the evaluation system based on the three domains of group work behaviors [24].

In the medical school of Mie University, undergraduate medical students learn preclinical subjects in their third and fourth grades. They participate in small group PBL activities with clinical scenarios in those preclinical years. The PBL sessions are intended to encourage students to discuss, extract learning tasks, and solve problems in group work. Moreover, these sessions aim to improve students’ learning attitude and communication skills. Students’ behaviors in these sessions are assessed by the peer evaluation system, which is the same as in the book review sessions. Our previous studies have confirmed the reliability of students in evaluating their peers by using that three-domain evaluation system in PBL sessions [25, 26].

Book review sessions and PBL sessions are operated in the scheme of groupworks and have similarities in the expected learning outcomes of teamwork, communication skills and learning attitude. As the same behaviors were assessed in both programs by peer evaluation, we decided to follow and analyze the scores of same students on defined parameters. The first research question in this study is to find the relationship of groupwork behaviors in liberal arts education and those in medical education by a follow-up analysis of peer evaluation scores. Some studies have reported the possible relationship of peer evaluation and academic achievement of medical students [27, 28]. Another research question in this study is to find the relationships of peer evaluations of students’ behavior in group work to their academic achievement.

We speculated that early exposure of students to group work evaluated by peers may be useful for their learning in upcoming years. This study was conducted to prove the positive relationships among groupwork behaviors in different years and academic achievement in preclinical years. We followed and analyzed the scores of same students from their first grade to preclinical years. The study aimed to clarify the importance of groupworks and to encourage students to participate well in their groupwork activities.

## Methods

### Study participants

We monitored the scores of a cohort consisting of medical students recruited in three consecutive years longitudinally (first grade students in 2015, 2016 and 2017). The students who participated in book review sessions in their first grade and PBL sessions and paper tests in the subsequent preclinical years were eligible for this study. Students from 2015 participated in book review sessions in 2015–2016. Then, they subsequently participated in PBL and paper tests in 2017–2018 and 2018–2019. Students from 2016 participated in book review sessions in 2016–2017 and PBL and paper tests in 2018–2019 and 2019–2020. Students from 2017 participated in book review sessions in 2017–2018 and PBL and paper tests in 2019–2020 and 2020–2021. All the students from the three cohorts participated in the book review sessions in their first grade and PBL and paper tests in their preclinical years. There was no modification of the groupwork programs (book review sessions and PBL) or paper test system during the study period. The learning contents, dynamics of the groupworks, evaluation system and criteria, and paper test system were not different among the programs for the three cohorts. Therefore, we analyzed the scores of the students from the three cohorts together in this study.

### Groupworks in book review sessions

**Fig. 1:**
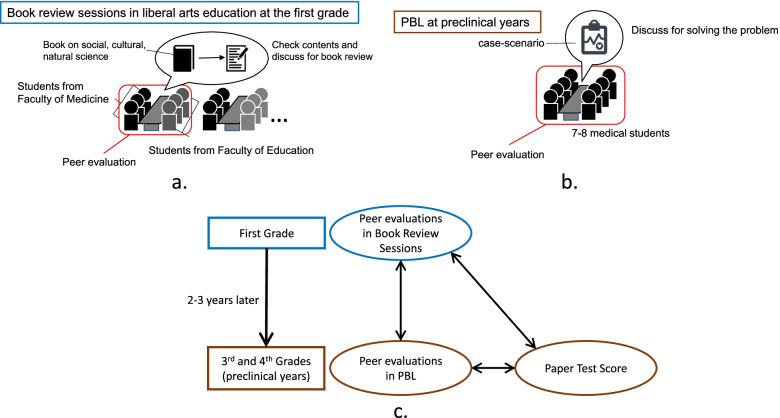
Groupworks in book review sessions and PBL sessions and data used for the correlation analyses. **a** Groupworks in book review sessions. **b** Groupworks in PBL sessions. **c** Data used for the correlation analyses

At Mie University, all first-grade students, including those from the Faculty of Medicine, participated in book review sessions of liberal arts education to attain generic skills (Fig. 1a). These sessions were implemented using the same peer evaluation system as in PBL sessions in the preclinical years of medical school. To communicate with various colleagues, small groups were organized with student members from at least two different schools. This arrangement aimed to develop students’ cooperativeness with members who have different backgrounds. For the medical students from the Faculty of Medicine, small groups in book review sessions were organized together with the students from the Faculty of Education. Each small group had four to six student members, including at least two medical students from the Faculty of Medicine and at least two students from the Faculty of Education (Fig. 1a). Each group carried out their group discussion on a book to be reviewed in 15 sessions. In the earlier sessions, students in each group selected a book to be reviewed. Standard guidelines for the books to be reviewed were provided to the students. The books eligible to be reviewed were those providing knowledge about natural science, social science, literature, or history. Fictions, novels, and how-to books were not eligible to be reviewed. The students in each group shared their opinions and discussed the selected book in the following sessions. Based on these discussions and opinions, each student prepared a book review report for the final session. The report was submitted to the teacher and distributed to group members.

Instructions about evaluating peers were given to students three times during book review sessions: at the 1st, 7th, and 14th sessions. The domains for evaluation were degree of prior learning, contribution to group discussion, and cooperative attitude with 10 points for each domain. The students were explained about the importance of peer evaluation and the evaluated behaviors in academic and career life. They were carefully instructed to make the evaluations only on the behaviors during the specific groupworks and not on the behaviors during other learning activities. Peer evaluations were made for a total of 15 book review sessions. Then, each student uploaded evaluations for self and other group members to Mie University Moodle, a web-based learning management system (Additional file 1, Additional file 2). Cumulative final score of 10 for these sessions was accessible to students at the end of 15 sessions. The final score was the combination book review score, which was given by teachers and students according to the quality of their book review report, and teachers’ evaluation and peer evaluation of groupwork behaviors. No student knew exactly how much he or she scored on peer evaluations.

### Groupworks in PBL at preclinical years

In the third and fourth grades of medical school, students carried out their group work in PBL sessions. There were 48 PBL sessions for 10 units organized into 5 modules (2 units in 1 module) (Additional file 3). There were approximately eight students in each group of PBL sessions (Fig. 1b). Members in the groups were organized randomly for the first module. In the following modules, the groups were reorganized for each module. Reorganization was based on the students’ paper test scores of the former unit of the previous module (Additional file 3). Therefore, there was a regular change in group members after each module. The aims of group reorganization were (1) to develop students’ ability to cooperate with any members, (2) to mix students of different academic achievements, and (3) to reduce social bias in peer evaluation. The last 2 units (Module 5) were conducted in a large room in which eight small groups were facilitated by a teacher. The groupworks in these units were similar to those in the previous units.

After each unit’s PBL sessions, the students uploaded peer evaluation scores, including self-evaluation scores, to Mie University Moodle (Additional file 1, Additional file 2, Additional file 3). The evaluation system was the same as that in the book review sessions. A paper test was held at the end of each unit (Additional file 3). After sitting for a paper test, each student received a unit report containing his or her average evaluation scores for the group work and the paper test score. The reported evaluations included teachers’ evaluations and peer evaluations separately. However, the individual peer evaluation score given by a student to another group member was not provided to the students. Therefore, peer evaluation was anonymous. No student knew how he or she had scored from a particular group member. Explanations and instructions about peer evaluation were given as in the book review sessions that they had participated in their first grade. Instructions for peer evaluation were given at the beginning of the PBL session of the first unit, and instruction manuals were provided. Moreover, the students could easily access the related information about the evaluation system on the university’s Moodle.

### Data analysis

Average scores of peer evaluations for book review sessions were used for analysis. For the former two academic years (2015 and 2016 intake students), the average peer evaluations for PBL sessions and the average paper test scores in all units (U1-U10) were used for analysis. For the latter academic year (2017 intake students), the average peer evaluations of PBL and average paper test scores in units 1 to 4 were used. This was because PBL sessions and paper tests in the remaining units (U5-U10) were changed to online-based programs according to preventive measures for coronavirus spread. The data were analyzed in R version 3.5.2 (R Core team 2018) and IBM SPSS 27.0 (IBM Corp. 2020). The scores of peer evaluations in the book review sessions, subsequent peer evaluation scores in PBL and paper test scores were analyzed for correlations in respective pairs (Fig. 1c). Spearman’s correlation coefficient was used to analyze the correlations among the scores. The relationships were interpreted according to the value of calculated correlation coefficients [29]. Comparisons were made between peer evaluation scores in the book review sessions and those in the subsequent PBL sessions. The Wilcoxon signed-rank test was used to compare the respective scores in the book review sessions and the subsequent PBL sessions. All analyses were conducted with two-sided tests at a significance level of 0.05.

## Results

Scores of 340 students in total were eligible for analysis: 113 students from 2015 intake, 107 students from 2016 intake and 120 students from 2017 intake.

The relationships among peer evaluation scores for the degree of prior learning in book review sessions, those in PBL, and paper test scores were assessed by correlation analysis (Additional file 4). The results showed that peer evaluation scores for the degree of prior learning in book review sessions were correlated with those in PBL sessions (r_s_ = 0.355, *p* < 0.001) (Table 1, Additional file 4). Paper test scores were found to be correlated with peer evaluation scores for the degree of prior learning in book review sessions (r_s_ = 0.338, *p* < 0.001) and to those in PBL sessions (r_s_ = 0.551, *p* < 0.001) (Table 2, Additional file 4).

**Table 1:** Correlations of peer evaluation scores for respective domains in book review sessions and PBL sessions (*n* = 340)

	Correlation coefficient (r_s_)	p
Degree of prior learning	0.355^a^	< 0.001
Contribution to group discussion	0.283^a^	< 0.001
Cooperative attitude	0.225^a^	< 0.001

^a^ significant correlation

**Table 2:** Correlations between peer evaluations in group work and paper test scores (*n* = 340)

	Book review sessions		PBL sessions	
	Correlation coefficient (r_s_) (to paper test score)	p	Correlation coefficient (r_s_) (to paper test score)	p
Degree of prior learning	0.338^a^	< 0.001	0.551^a^	< 0.001
Contribution to group discussion	0.146^a^	0.007	0.515^a^	< 0.001
Cooperative attitude	0.217^a^	< 0.001	0.412^a^	< 0.001

^a^ significant correlation

The relationships among peer evaluation scores for contribution to group discussion in book review sessions, those in PBL sessions, and paper test scores were assessed by correlation analysis (Additional file 5). There was a significant correlation between peer evaluation scores for contributions to group discussions in book review sessions and those in PBL sessions (r_s_ = 0.283, *p* < 0.001) (Table 1, Additional file 5). Paper test scores were found to be correlated with peer evaluation scores for contribution to group discussion in book review sessions (r_s_ = 0.146, *p* = 0.007) and to those in PBL sessions (r_s_ = 0.515, *p* < 0.001) (Table 2, Additional file 5).

The relationships among peer evaluation scores for cooperative attitude in book review sessions, those in PBL sessions, and paper test scores were assessed by correlation analysis (Additional file 6). The results showed a significant correlation between peer evaluation scores for cooperative attitudes in book review sessions and those in PBL sessions (r_s_ = 0.225, *p* < 0.001) (Table 1, Additional file 6). Paper test scores also correlated with peer evaluation scores for cooperative attitudes in book review sessions (r_s_ = 0.217, p < 0.001) and to those in PBL sessions (r_s_ = 0.412, p < 0.001) (Table 2, Additional file 6).

The peer evaluation scores for the degree of prior learning in PBL were found to be significantly higher than those in book review sessions (Z = − 8.881, *p* < 0.001) (Additional file 7). Comparing peer evaluations for contribution to group discussion, the PBL scores were significantly higher than those in book review sessions (Z = − 5.331, *p* < 0.001) (Additional file 7). The scores of cooperative attitudes during groupworks in PBL sessions were found to be significantly higher than those in book review sessions (Z = − 10.363, *p* < 0.001) (Additional file 7).

## Discussion

The present study revealed a positive correlation between peer evaluation scores of group work in the first grade of medical school and those in the subsequent years. A significant correlation was found between scores of peer evaluations for degree of prior learning in book review sessions and those in PBL sessions (Table 1, Additional file 4). The students carried out prior learning for book review sessions by preparing the relevant literature of the selected books. In their subsequent preclinical years, they prepared for PBL sessions by searching the information required for understanding and solving the given problem. Despite the differences in preparatory tasks for group work, medical students’ practice of preparation for group work in the early school years was related to that in their subsequent preclinical years. Preparation for a particular task is an important competency for a medical professional. Our findings mean that students who prepare properly for group work in early school years will probably prepare well for group work in subsequent schoolyears.

Book review sessions in the early school and PBL sessions in the subsequent preclinical years had different topics for discussion. In the book review sessions, based on the topic of the selected book, students may have varying interests in the group work. Some medical students who wanted to study only medical topics may be less active in these sessions. Another situation that may affect the difference in communication behaviors between book review sessions and PBL sessions was the heterogeneity of group members in book review sessions. In these sessions, each group was organized intermixing the students from the Faculty of Medicine and Faculty of Education. However, the scores for contribution to group discussion in book review sessions were found to be correlated to those in PBL of the subsequent years (Table 1, Additional file 5). Our findings revealed the relationship between the scores of two group discussion behaviors in different school years in the cohort. If students have active discussion behaviors in the early years of medical school, good communication skills can probably be expected in the following years. Moreover, their learning may be improved, as discussion among students has been reported to have positive effects on their learning [30]. The communication skill of medical students was suggested to be acquired by observing others and exercising the skill with appropriate feedback [31]. It was also reported that emphasizing communication skills during clinical training could encourage students to practice better communication in their career life [31].

The scores for cooperative attitude in the groupworks were significantly correlated (Table 1, Additional file 6). The students seemed to maintain their cooperative attitude to some degree for years. Although the relationship is weak, we believe that emphasizing cooperative group work in the early years of medical school can improve students’ groupwork behaviors. It will probably help them to be more cooperative in following years. Similar to our suggestion, cooperative learning was proposed to be encouraged in the early years of medical school, as it may be helpful for the development of interpersonal skills needed for health care professionals [32].

In comparison, peer evaluation scores in the respective domains were found to be higher in the PBL sessions than in the book review sessions (Additional file 7). The students seemed to behave better in preclinical years’ PBL sessions than in the book review sessions of their first grade. The practice of groupworks in the book review sessions may contribute to the improvement of the behaviors in the PBL sessions. Preference to study medical science could also affect the difference in behaviors between the book review sessions and the subsequent PBL sessions. Convenience to work together with group members who had been in the same class for years may be another reason for having higher scores in the PBL sessions. Arranging early years’ group work with interesting content, which can attract the attention of medical students, may enhance their active participation. Consequently, their groupwork behaviors, which can be carried over to the following years of medical school, will probably be improved.

Another significant finding in this study is the relationship between peer evaluation scores of the groupworks and preclinical years’ paper test scores. Paper test scores correlated with peer evaluation scores for the degree of prior learning in book review sessions and with those in PBL sessions (Table 2, Additional file 4). The students’ academic achievement was significantly related to their practice of preparation for groupworks in the same year and even to that in the early school year. This means that we could expect good academic achievement from the students who prepared well for their groupworks. It was similarly reported that appropriate class preparation by students could help to control their anxiety and improve their academic achievement [33].

The correlation between peer evaluation scores for contribution to group discussion in book review session and paper test scores was very weak or negligible (Table 2, Additional file 5). Peer evaluations of contribution to group discussion in PBL correlated significantly with paper test scores (Table 2, Additional file 5). Although the correlation was weaker than that of the degree of prior learning, the contribution to group discussion was significantly related to the students’ academic achievement. Good academic achievement could be expected from the students who discussed actively in their learning activities. A study about medical students’ performance similarly reported that participation in PBL sessions was associated with better academic performance [34]. Students’ social and communication skills, which are required for active group discussion, were reported to be related to their academic performance [28, 35]. Moreover, active participation in class was supposed to improve the academic achievement of students by controlling their anxiety [33].

Paper test scores were found to be correlated with the scores for cooperative attitude in book review sessions and to those in PBL sessions (Table 2, Additional file 6). We can expect good academic achievement from the students who participated cooperatively in their group work. A previous study also reported that cooperative group learning could improve the academic achievement of students [36].

According to the results, we can interpret that medical students’ peer evaluations in groupworks were related to their academic achievement. Even the evaluations from early years’ group work had some degree of positive correlations with academic achievement in the subsequent years. The findings agree with previous studies that reported a positive relationship between peer evaluation and the academic performance of medical students [27, 28]. We suppose that emphasizing teamwork trainings in the early years of medical school can improve students’ academic and professional life. Additionally, peer evaluation should be implemented in those trainings, as it has been suggested to improve students’ learning and academic performance [37–39].

Mie University emphasizes the importance of teamwork and communication skills from the first grade. Peer evaluation for group work is introduced in the first grade because its early practice is suggested to be beneficial for students’ learning [18]. Furthermore, the same system of peer evaluation is used in the groupworks of different schoolyears. In both book review sessions and PBL sessions, detailed instructions for peer evaluation were provided to the students. Such instructions have been suggested to strengthen the reliability of the evaluations [15]. A worrisome bias in peer assessments or evaluations is caused by the social relationships of group members [40]. One possible solution to reduce such bias is to use anonymous evaluations, which is considered essential in peer evaluations [41, 42]. In both book review sessions and PBL sessions, the students were instructed to avoid social bias and to maintain anonymity in providing evaluations.

There are some limitations in this study. First, the study was conducted in a single medical school. Second, the group work in the book review sessions may be unique to Mie University. Third, the peer evaluation process in the book review sessions may be the students’ first experience of judging peers, and it may affect the accuracy of the evaluations. Fourth, despite the instructions, some students’ evaluations may be confused with judgments on behaviors in other learning activities and daily campus life. These limitations may affect the generalizability of the results. Using the same evaluation system to assess the three important competencies, maintaining anonymity of peer evaluations, and providing proper instructions for the evaluations enhanced the reliability of the study and its generalized applicability. As this study is an observational study, we could not conclude that the correlations among the respective group work and academic achievement were cause-and-effect relationships. Future studies across multiple medical schools and extending the study to clinical years may provide more useful information.

In conclusion, the present longitudinal study has revealed the relationships between group work behavior scores in two different academic years of medical education. The relationships between those behavior scores and academic achievement were also revealed in this study. Even groupwork behaviors in the early school year were found to be related to academic achievement in the subsequent preclinical years to some degree. For every medical school, academic achievement is a goal, and teamwork and communication skills are important competencies. Furthermore, students’ motivation to study is suggested to have a great influence on their performance and academic achievement [34, 43]. Realizing the importance of their learning activities may motivate them. The results in this study highlighted the importance of teamwork trainings and peer evaluation. With close monitoring of students’ peer evaluation scores, teachers can provide appropriate support to students with below average peer evaluations. Then, the students will be able to improve their teamwork and academic achievement. To our knowledge, there is limited information about longitudinal studies on peer evaluations of small group learning in general education and medical education. This study may provide useful information for teamwork trainings in different years of medical education curriculum.

## Supplementary Information

**Additional file 1.**

**Additional file 2.**

**Additional file 3.**

**Additional file 4.**

**Additional file 5.**

**Additional file 6.**

**Additional file 7.**

## Data Availability

The datasets used and/or analyzed during the current study are available from the corresponding author on reasonable request. In Mie University, the scores of students are considered as confidential information. Therefore, the data set cannot be deposited publicly.

## Abbreviation

PBL: Problem-based learning
