# Supplementary material for: Peer evaluations of group work in different years of medical school and academic achievement: how are they related?
Source: BMC Med Educ. 2022 Feb 16;22:102. doi: 10.1186/s12909-022-03165-5 (PMC8851726; doi:10.1186/s12909-022-03165-5)
Supplement: Supplementary file 1 — Additional file 1. [file 12909_2022_3165_MOESM1_ESM.docx]

**Providing peer evaluation for the group members** (translated from Japanese)

Please make the evaluations exclusively on the groupwork. Do not evaluate according to personal relationships, academic grades, and performance and attitudes in other learning activities. The evaluation scores will be calculated based on the average scores of the evaluations provided by peers. Individual evaluations are anonymized and will not be disclosed.

**1. Degree of Prior learning**

Evaluate each of your group members on how much he/she had prepared for the groupwork (Figure S1). Be sure to make honest evaluations. The evaluation is based on 10 points in which 10 means perfect. Pass mark is 6. If you make the evaluation less than 6, please provide the reason for the evaluation. Choose N/A if the member was absent in all of the sessions.

**2. Contribution to group discussions**

Evaluate each of your group members on how much he/she had contributed to the improvement of the knowledge and thinking of other group members through discussion during the groupwork (Figure S1). Be sure to make honest evaluations. The evaluation is based on 10 points in which 10 means perfect. Pass mark is 6. If you make an evaluation less than 6, please provide the reason for the evaluation. Choose N/A if the member was absent in all of the sessions.

**3. Cooperative attitude**

Evaluate each of your group members on how much his/her cooperativeness had contributed to improve the environment for group learning (Figure S1). Be sure to make honest evaluations. The evaluation is based on 10 points in which 10 means perfect. Pass mark is 6. If you make an evaluation less than 6, please provide the reason for the evaluation. Choose N/A if the member was absent in all of the sessions.

**Figure legends (for figures in Additional files)**

**Figure S1 (Additional file 2).** Example of user interface for peer evaluation on Mie University Moodle (translated from Japanese)

**Figure S2 (Additional file 4)**. Relationships among peer evaluation scores for degree of prior learning and paper test score

BRS_Q1 – peer evaluation score for degree of prior learning in book review sessions of liberal arts education in the first grade of medical school

PBL_Q1 – peer evaluation score for degree of prior learning in problem-based learning sessions in the subsequent preclinical years

A – scatter plot for BRS_Q1 and PBL_Q1; X-axis – BRS_Q1, Y-axis – PBL_Q1

B – scatter plot for BRS_Q1 and Paper Test Score; X-axis – BRS_Q1, Y-axis – Paper Test Score

C – scatter plot for PBL_Q1 and Paper Test Score; X-axis – PBL_Q1, Y-axis – Paper Test Score

***, *p* < 0.001

**Figure S3 (Additional file 5).** Relationships among peer evaluation scores for contribution to group discussion and paper test score

BRS_Q2 – peer evaluation score for contribution to group discussion in book review sessions of liberal arts education in the first grade of medical school

PBL_Q2 – peer evaluation score for contribution to group discussion in problem-based learning sessions in the subsequent preclinical years

A – scatter plot for BRS_Q2 and PBL_Q2; X-axis – BRS_Q2, Y-axis – PBL_Q2

B – scatter plot for BRS_Q2 and Paper Test Score; X-axis – BRS_Q2, Y-axis – Paper Test Score

C – scatter plot for PBL_Q2 and Paper Test Score; X-axis – PBL_Q2, Y-axis – Paper Test Score

**, *p* < 0.01; ***, *p* < 0.001

**Figure S4 (Additional file 6).** Relationships among peer evaluation scores for cooperative attitude and paper test score

BRS_Q3 – peer evaluation score for cooperative attitude in book review sessions of liberal arts education in the first grade of medical school

PBL_Q3 – peer evaluation score for cooperative attitude in problem-based learning sessions in the subsequent preclinical years

A – scatter plot for BRS_Q3 and PBL_Q3; X-axis – BRS_Q3, Y-axis – PBL_Q3

B – scatter plot for BRS_Q3 and Paper Test Score; X-axis – BRS_Q3, Y-axis – Paper Test Score

C – scatter plot for PBL_Q3 and Paper Test Score; X-axis – PBL_Q3, Y-axis – Paper Test Score

***, *p* < 0.001

**Figure S5 (Additional file 7).** Comparison of the respective peer evaluation scores

a. Comparison of the peer evaluation scores for degree of prior learning

BRS_Q1 – peer evaluation score for degree of prior learning in book review sessions of liberal arts education in the first grade of medical school

PBL_Q1 – peer evaluation score for degree of prior learning in problem-based learning sessions in the subsequent preclinical years

b. Comparison of the peer evaluation scores for contribution to group discussion

BRS_Q2 – peer evaluation score for contribution to group discussion in book review sessions of liberal arts education in the first grade of medical school

PBL_Q2 – peer evaluation score for contribution to group discussion in problem-based learning sessions in the subsequent preclinical years

c. Comparison of the peer evaluation scores for cooperative attitude

BRS_Q3 – peer evaluation score for cooperative attitude in book review sessions of liberal arts education in the first grade of medical school

PBL_Q3 – peer evaluation score for cooperative attitude in problem-based learning sessions in the subsequent preclinical years
