# Supplementary figures and images for: Peer evaluations of group work in different years of medical school and academic achievement: how are they related?
Source: BMC Med Educ. 2022 Feb 16;22:102. doi: 10.1186/s12909-022-03165-5 (PMC8851726; doi:10.1186/s12909-022-03165-5)

## Slide 1
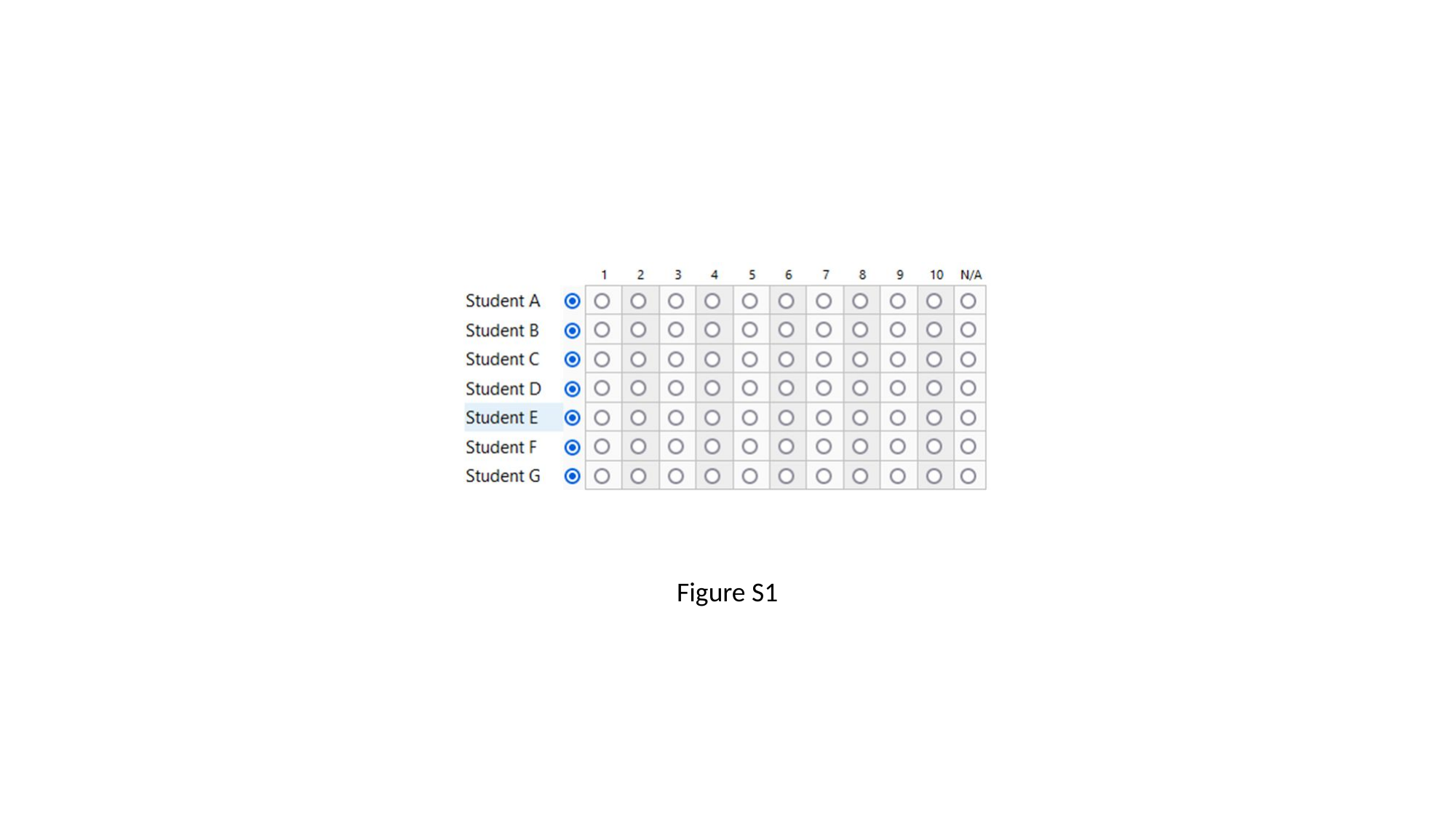

Figure S1

Supplement: Supplementary file 2 — Additional file 2. [file 12909_2022_3165_MOESM2_ESM.pptx]

## Slide 1
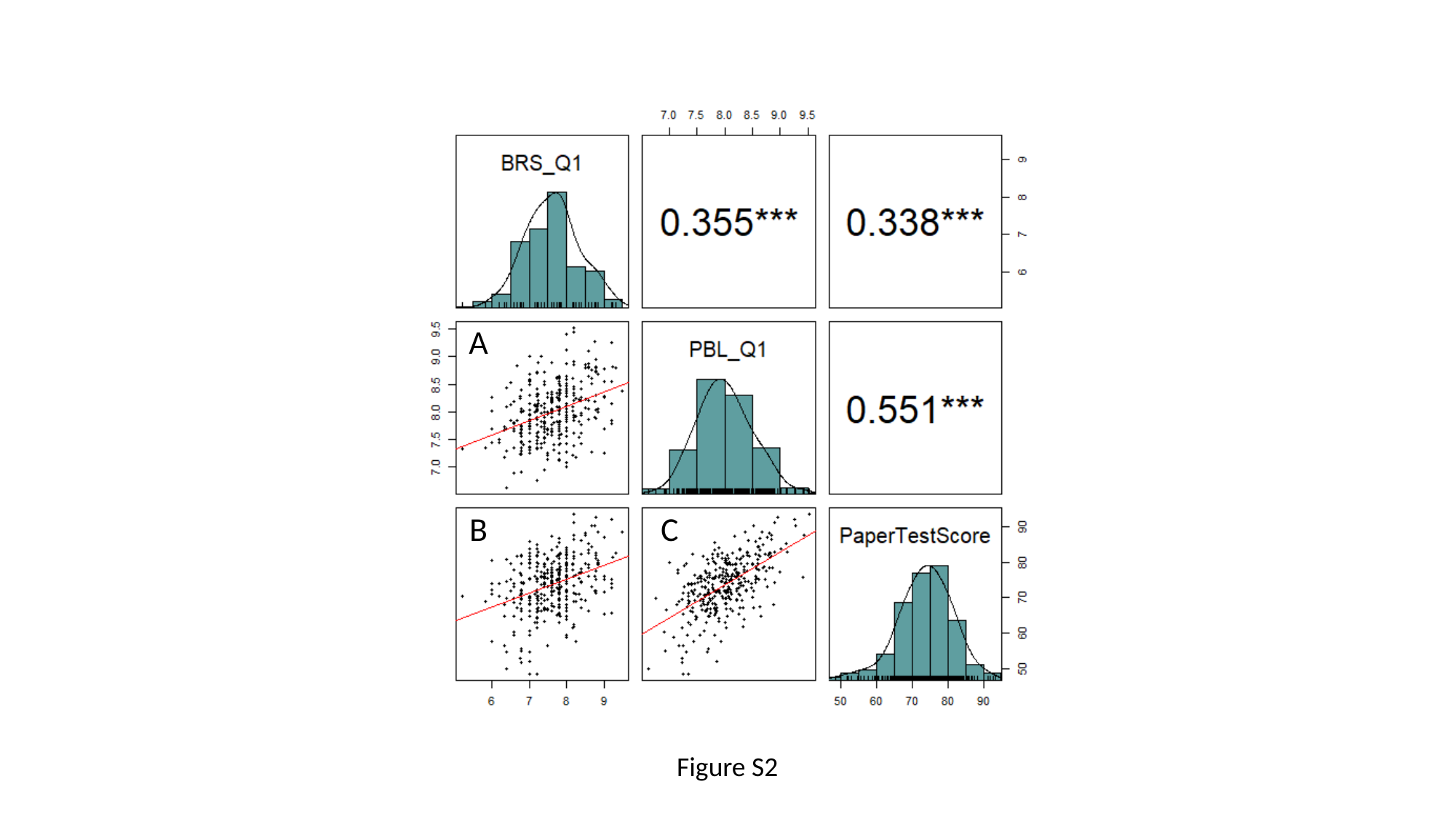

A
C
B
Figure S2

Supplement: Supplementary file 4 — Additional file 4. [file 12909_2022_3165_MOESM4_ESM.pptx]

## Slide 1
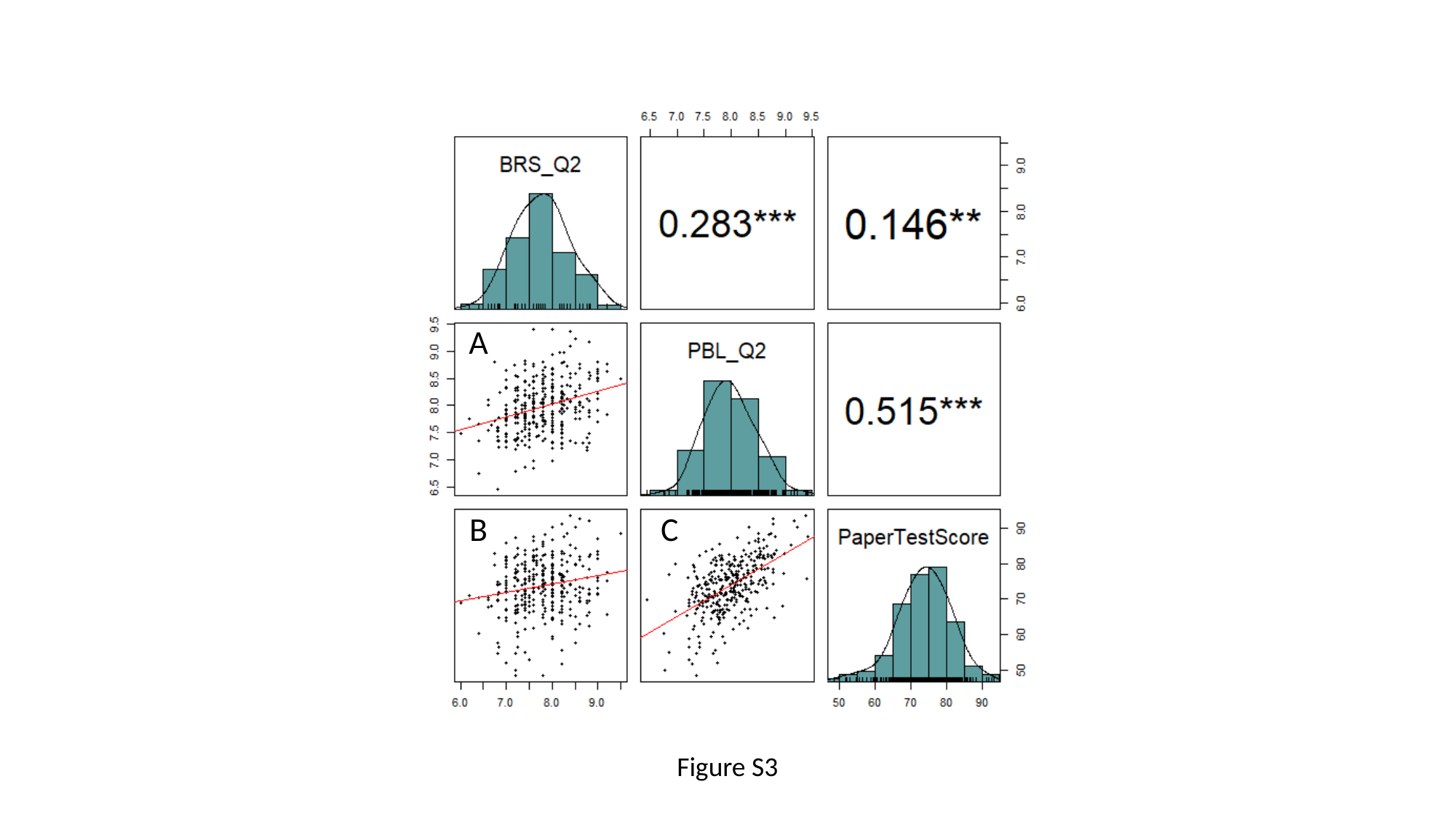

A
C
B
Figure S3

Supplement: Supplementary file 5 — Additional file 5. [file 12909_2022_3165_MOESM5_ESM.pptx]

## Slide 1
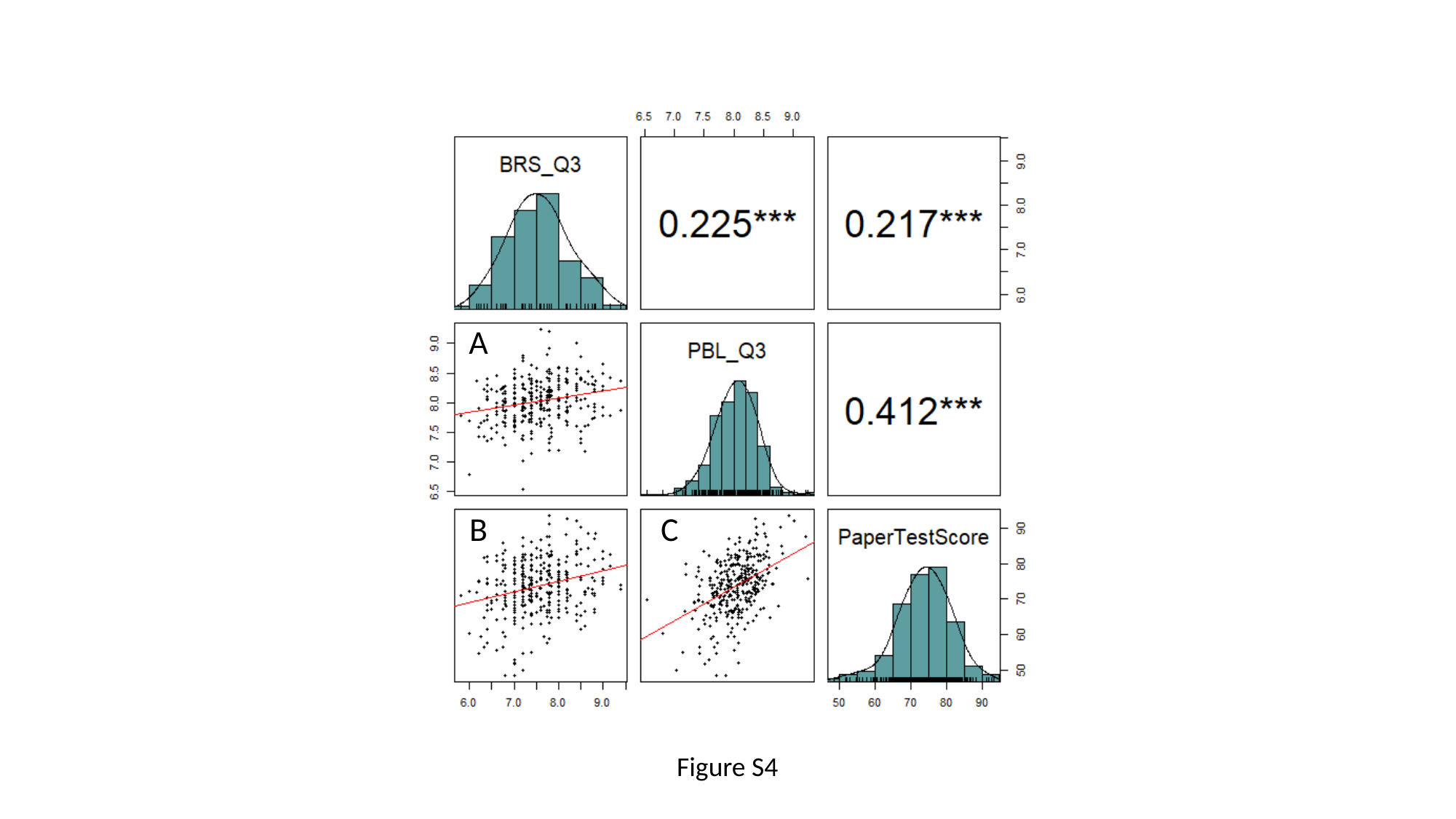

A
C
B
Figure S4

Supplement: Supplementary file 6 — Additional file 6. [file 12909_2022_3165_MOESM6_ESM.pptx]

## Slide 1
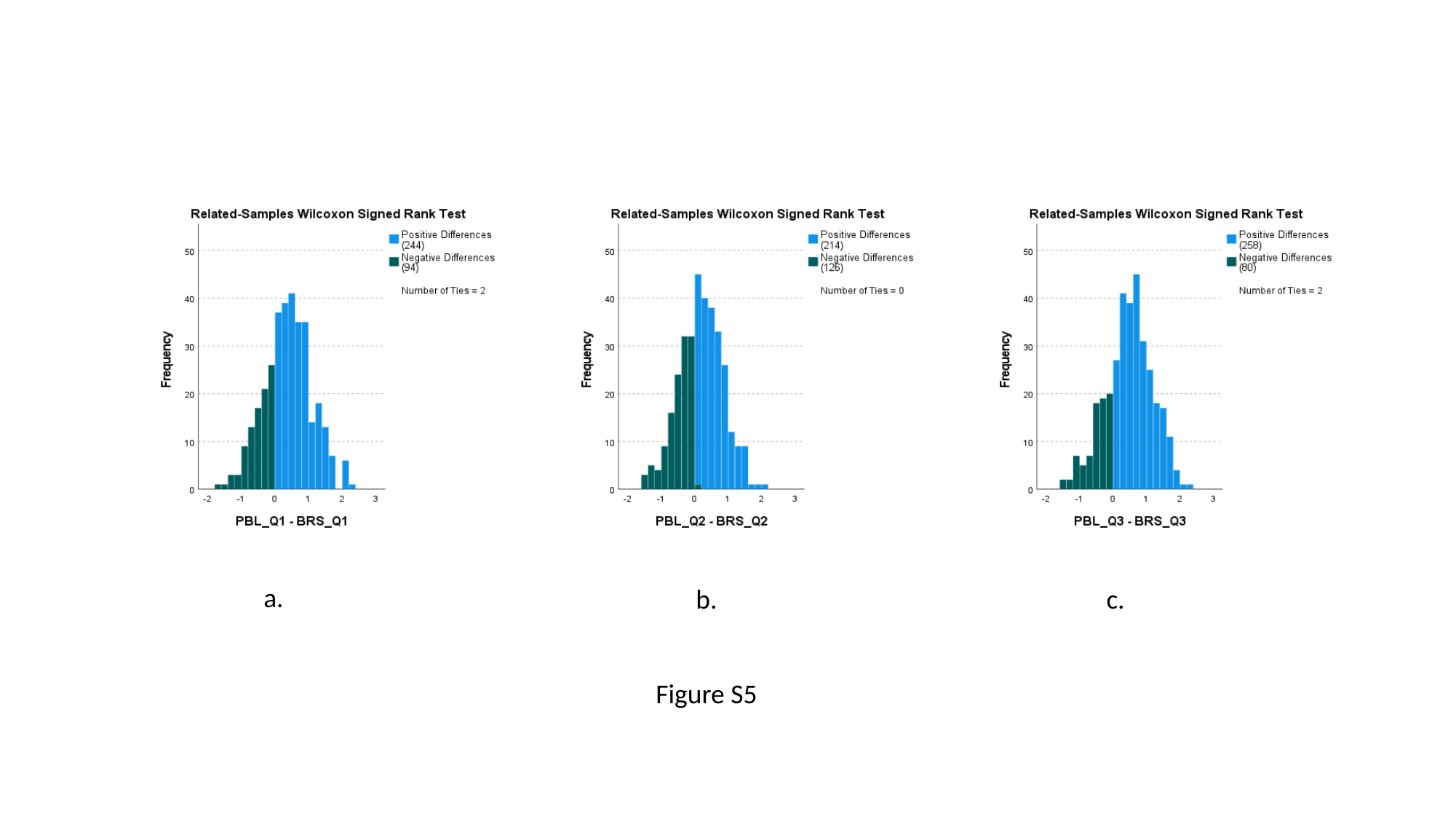

a.
b.
c.
Figure S5

Supplement: Supplementary file 7 — Additional file 7. [file 12909_2022_3165_MOESM7_ESM.pptx]
