# Supplementary material for: Peer evaluations of group work in different years of medical school and academic achievement: how are they related?
Source: BMC Med Educ. 2022 Feb 16;22:102. doi: 10.1186/s12909-022-03165-5 (PMC8851726; doi:10.1186/s12909-022-03165-5)
Supplement: Supplementary file 3 — Additional file 3. [file 12909_2022_3165_MOESM3_ESM.docx]

Table A1. Arrangement of PBL sessions and paper tests

| Grade | Module | Unit | Learning contents |
| --- | --- | --- | --- |
| Third Grade | M1 | U1 | Infection, Immunology ● ◊ |
|  |  | U2 | Cardiovascular system, Hematology ● |
|  | M2 | U3 | Respiratory system, Endocrinology, Metabolism ● ◊ |
|  |  | U4 | Kidney, Urinary tract ● |
|  | M3 | U5 | Neurology ● ◊ |
| Fourth Grade |  | U6 | Digestive system ● |
|  | M4 | U7 | Breast, Female reproductive system ● ◊ |
|  |  | U8 | Pediatrics, Geriatric medicine ● |
|  | M5 | U9 | Emergency medicine, Ear, Nose, Throat, Skin, Eye ● |
|  |  | U10 | Orthopedics, Maxillofacial surgery, Anesthesiology, Psychiatric medicine, Family medicine ● |

● Teacher and peer evaluation and paper test; ◊ Paper test score of the unit that would be used to reorganize the small groups for the next module
